# Diageotropica and Lateral Rooting, the Rest of the Story

**DOI:** 10.3389/fpls.2016.01239

**Published:** 2016-08-22

**Authors:** Richard W. Zobel

**Affiliations:** Plant Science Research Unit, Agricultural Research Service, US Department of AgricultureRaleigh, NC, USA

**Keywords:** ethylene, auxin, dgt mutant, diageotropica, cyclophilin

Plant roots have long been of practical and scientific interest for their role in plant stature, and water and nutrient uptake. One of the first classes of root to be singled out and explored were the lateral roots. Much of the study of lateral roots has been with seedling plants due to their early development and relatively easy access. McCully (1975) and Charlton (1991) have reviewed lateral root initiation and out growth with a focus on anatomy and patterns. Torrey (1976) reviewed the physiological control of lateral root initiation, and Ormon-Ligeza et al. (2013) provide a more recent molecular review. Lateral root initiation in *Arabidopsis* has been described anatomically (Dubrovsky et al., 2000, 2006) and some of the related molecular aspects (Ivanchenko et al., 2006) have been studied, leading to eventual description of the pathways of control of lateral root initiation and outgrowth. One of the tools used in the study of lateral root initiation and its control has been plant mutants, which lack or have modified patterns of lateral root initiation. One such mutant, *diageotropica* (*dgt*) has recently been used to characterize a non-auxin compound, which is required for the localization of lateral root initiation in the pericycle (Ivanchenko et al., 2015; Spiegelman et al., 2015). These recent results suggest that the pathway involved is conditioned by a cyclophilin which is produced in the shoot and transported to the root where it effects the localization of auxin induced cellular division of pericycle cells in a parent root (Ivanchenko et al., 2015; Spiegelman et al., 2015). But is that the end of the story?

As Retzer and Luschnig (2015) and Su et al. (2015) suggest, the identification and role of the mutant cyclophilin adds an interesting twist to the auxin signaling story. However, there is an additional complicating twist that has been all but ignored because of early attempts to discredit an initial hypothesis. The original mutant (diageotropica, *dgt* or *dgt1-1*) is a single gene point mutation resulting in an amino acid substitution (G_137_ changed out) in a protein called a cyclophilin (Oh et al., 2002, 2006). This mutation, though relatively simple, conditions a series of pleiotropic phenes (see Lynch and Brown, 2012, for a definition of a phene) resulting in a complex phenotype: lack of lateral rooting, diageotropic growth habit of roots, and shoots, folded (curled) leaves and cotyledons, open hypocotyl hook, and modified vascular system (Zobel, 1973, 1974; Madlung et al., 1999).

Zobel (1972, 1973, 1974) first discovered, and characterized the mutant (*dgt*) genetically, morphologically and physiologically. Zobel's conclusion was that, due to the total recovery of a normal phenotype with the application of trace amounts of ethylene [5 nl l^−1^—i.e., catalytic, not physiological (100 nl l^−1^), level concentrations; 5 nl l^−1^ is not the lowest level found to be effective, but is the lowest level reported] to the shoot, the mutant was an ethylene requiring mutation. Zobel's research demonstrated an abnormal pattern of wound ethylene development (up to 20 h rather than the typical 6 h), and normal fruit ripening and ethylene production with floral production and fruit ripening. Jackson (1979), Bradford and Yu (1980), and Kelly and Bradford (1986) carried out experiments on the *dgt* mutant without using ethylene gas [i.e., not assessing Zobel's (1973, 1974) ethylene methodology or results]. The first paper of this series (Jackson, 1979) asked the question “Is the diageotropic tomato ethylene deficient?.” Jackson (1979) duplicated much of Zobel's non-ethylene application results while assuming that, in the mutant, wound ethylene production finished within the classical 6 h. His assumption was not supported by either his or Zobel's presented data (greater than 8 and 20 h duration of wound ethylene production in non-leaf tissues, respectively), thus invalidating Jackson's methodology and conclusions. The other two papers also had evidence of long term wound ethylene production from distinctly different tissues. Since low ethylene levels “normalized” the mutant, research with excised *dgt* tissues (i.e., tissues with stimulated wound ethylene production) cannot be relied on to give germane results. These three papers, using excised tissues, then claimed the mutation was an auxin insensitive mutation, not ethylene requiring, despite many normal responses to auxin, including normal auxin levels, normal auxin transport, flowering and fruiting, as well as other aspects of auxin mediated growth and development.

These conclusions attracted the attention of auxin researchers, especially the Lomax group (Hicks et al., 1989; Muday et al., 1995; Niebenfuhr et al., 2000; Oh et al., 2002; and additional), who, initially focused on auxin control of gravitropism. Except for the research of Madlung et al. (1999), none of the research with *dgt* has investigated the response to low levels of ethylene, but has focused on the apparent specific auxin insensitive nature of the mutant, with a strong effort on the lateral rooting aspect (Ivanchenko et al., 2006, 2015). Since Oh et al. (2006) demonstrated that a wild type cyclophilin complemented *dgt* plant has a normal phenotype, and Zobel (1973) and Madlung et al. (1999) demonstrated that ethylene (5 nl l^−1^) treated plants also have a normal phenotype, a critical question is: what is the actual relationship between the *dgt* gene product and ethylene? In other words, how can ethylene, at such a low concentration, overcome the effect (or rather lack of effect) of a mutant gene product?

Oh et al. (2006) suggest, from a lack of mutant cyclophilin in the hypocotyl, that the *dgt* mutants are null mutants. Spiegelman et al. (2015), Ivanchenko et al. (2015), and Zobel (1973) suggest that the cyclophilin is produced only in the leaves and that the mutant cyclophilin may not be mobile, thus questioning the hypothesis of null mutants. The ability of ethylene to “normalize” *dgt* suggests that it is not a null mutation, but rather an ineffective mutant. A rather simple minded hypothesis would be that ethylene facilitates folding of the mutant cyclophilin (see Su et al., 2015, and Jing et al., 2015), while still in the shoot, such that it becomes effective, and able to transport to the roots or other sites of action. The actual answer, when discovered, will impact the current concepts about auxin/ethylene interaction, and especially about the mechanism(s) of fine level ethylene action. In short, a revisiting of ethylene actions and auxin/ethylene crosstalk (Muday et al., 2012) with research using this mutant and ethylene levels at or below the nl l^−1^ level, is mandated. The *dgt* mutant and the host of *Arabidopsis* genes similarly involved (Ivanchenko et al., 2006) are the perfect tools for this effort.

## Author contributions

The author confirms being the sole contributor of this work and approved it for publication.

## Funding

All funding was from base funding from the USDA-ARS.

### Conflict of interest statement

The author declares that the research was conducted in the absence of any commercial or financial relationships that could be construed as a potential conflict of interest. The reviewer SH declared a shared affiliation, though no other collaboration, with the author RZ to the handling Editor, who ensured that the process nevertheless met the standards of a fair and objective review.
